# Primary parotid gland lymphoma: a case report

**DOI:** 10.1186/1752-1947-5-380

**Published:** 2011-08-15

**Authors:** Petros Konofaos, Eleftherios Spartalis, Paraskevas Katsaronis, Grigorios Kouraklis

**Affiliations:** 1 2nd Department of Propedeutic Surgery, 'LAIKO' General Hospital 17, Ag Thomas Street, Athens 11527, Greece

## Abstract

**Introduction:**

Mucosa associated lymphoid tissue lymphomas are the most common lymphomas of the salivary glands. The benign lymphoepithelial lesion is also a lymphoproliferative disease that develops in the parotid gland. In the present case report, we describe one case of benign lymphoepithelial lesion with a subsequent low transformation to grade mucosa associated lymphoid tissue lymphoma appearing as a cystic mass in the parotid gland.

**Case presentation:**

A 78-year-old Caucasian female smoker was referred to our clinic with a non-tender left facial swelling that had been present for approximately three years. The patient underwent resection of the left parotid gland with preservation of the left facial nerve through a preauricular incision. The pathology report was consistent with a low-grade marginal-zone B-cell non-Hodgkin lymphoma (mucosa associated lymphoid tissue lymphoma) following benign lymphoepithelial lesion of the gland.

**Conclusions:**

Salivary gland mucosa associated lymphoid tissue lymphoma should be considered in the differential diagnosis of cystic or bilateral salivary gland lesions. Parotidectomy is recommended in order to treat the tumor and to ensure histological diagnosis for further follow-up planning. Radiotherapy and chemotherapy should be considered in association with surgery in disseminated forms or after removal.

## Introduction

Mucosa associated lymphoid tissue (MALT) lymphomas are non-encapsulated clusters of lymphocytes found throughout the mucosal tissues of the aero-digestive tract. The non-Hodgkin type lymphomas that arise from these lymphocyte aggregates (MALT lymphoma) are of B-cell lineage, the commonest involving the salivary glands [1]. A MALT lymphoma has been presumed to be associated with autoimmune or inflammatory diseases [2]. The benign lymphoepithelial lesion (BLL) is also a lymphoproliferative disease that develops in the parotid gland. Although, BLL is a benign disease, subsequent malignancies have also been reported [3,4].

The lymphoma arising from MALT was first described by Isaacson and Wright in 1983 [5]. MALT lymphoma arising from salivary glands is a rare entity; available data in the literature are scarce, confined to small series and isolated case reports. The characteristics and clinical outcome of this unusual presentation are largely unknown [6]. Early diagnosis relies on a high index of suspicion.

In the present case report, we describe one case of BLL with a subsequent low transformation to grade MALT lymphoma appearing as a cystic mass in the parotid gland.

## Case Presentation

A 78-year-old Caucasian female smoker was referred to our clinic with a non-tender left facial swelling that had been present for approximately three years. The patient was otherwise asymptomatic. She had no history of malignancy or autoimmune diseases. A firm mobile mass was present in the left parotid gland.

Examination revealed a 5 cm firm mobile mass in the superficial lobe of the left parotid. The left facial nerve was intact (House-Brackmann scale Grade I). No other abnormalities were found in the nasopharynx, oral cavity, larynx or ears. There was no pathological enlargement of the cervical lymph nodes. Laboratory tests were within normal limits. Hepatitis B virus (HBV) and hepatitis C virus (HCV) serologies were negative.

**Figure 1:**
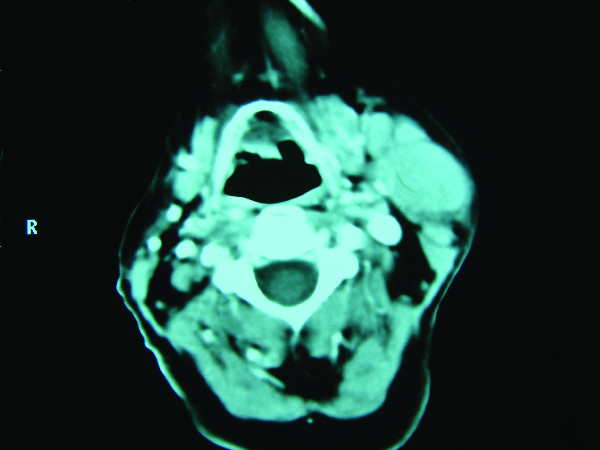
**Preoperative CT scan of the tumor of the left parotid gland**.

The patient had undergone ultrasonography-guided fine needle aspiration of the left parotid gland several months before. The cytological examination revealed mononuclear and inflammatory cells and a diagnosis of a chronic parotiditis was made. The patient was given antibiotics but with little effect. Unfortunately, the mass on the left parotid continued to enlarge. After admission to our clinic, a helical CT scan was performed which revealed a solid mass with an irregular surface in the left parotid gland (Figure 1).

**Figure 2:**
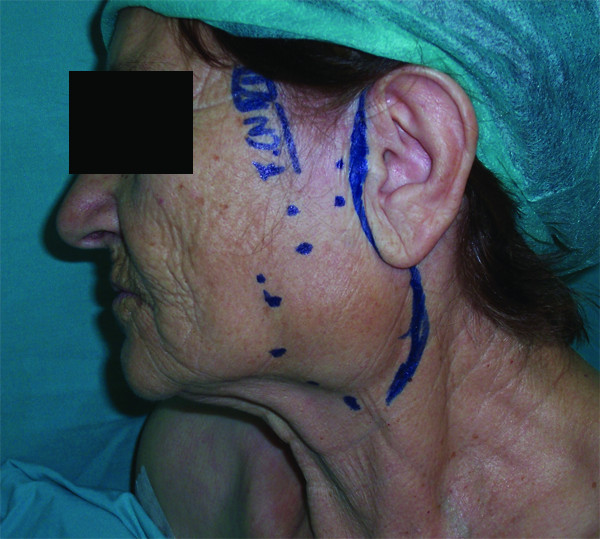
**Preoperative planning of the preauricular incision - the circular dotted line represents the tumors margins**.

**Figure 3:**
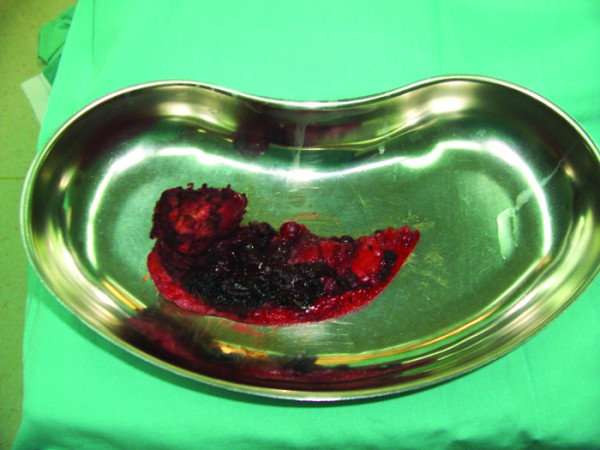
**The surgical specimen**.

The patient underwent resection of the left parotid gland with preservation of the left facial nerve through a preauricular incision (Figure 2). Identification of the branches of the facial nerve was made by using loupes magnification and with intraoperative electric stimulation of the identified branches of the facial nerve. The size of the resected tumor was 5 × 7 cm (Figure 3). The surgical specimen was sent for a histopathological examination.

The pathology report was consistent with a low-grade marginal-zone B-cell non-Hodgkin lymphoma (MALT lymphoma) following BLL of the gland. According to the report, there was infiltration of the normal salivary tissue by a heterogenous mixture of lymphocytes and isolated blastic cells.

Postoperative recovery was uneventful and facial nerve function was intact. CT scan of the head, neck, chest and abdomen at two and six months after the surgery revealed no evidence of lymphoma infiltration. Our patient had isolated surgical treatment without chemotherapy. By the time this report was completed, the patient had been followed for 13 months without evidence of recurrence.

## Discussion

Primary lymphomas of the salivary glands are rare and account for 4.7% of lymphomas at all sites [7]. A non-Hodgkin lymphoma of a salivary gland may appear as a painless, progressively enlarging mass [8-11]. Therefore, it is rarely suspected before biopsies or surgical removal. MALT lymphomas developing within the salivary glands may be related to chronic lymphoid hyperplasia.

The native absence of MALT within the salivary glands necessitates the development of acquired MALT from underlying lymphoid stimulation and infiltration before MALT lymphoma can develop [12]. Low-grade MALT lymphomas of the parotid gland usually arise in a setting of BLL [13]. The main histological characteristic of BLL of the parotid gland is the presence of clustered B cells inter-digitating with ductal epithelial cells. According to Amft et al [14], BLL can be considered a 'premalignant lesion' due to the fact that it can contain clonal populations of B cells, although it is generally regarded as a benign lesion. The transformation from BLL to MALT lymphoma is believed to be a multi-step process. The initial event of this process may be a long-term stimulation of activating B cells by an inflammatory stimulus [15]. Hiltbrand et al [16] also suggested that MALT lymphomas arise from BLL, not from intraparotid lymphoid aggregates.

Association between MALT lymphoma and autoimmune diseases such as systemic lupus erythematous [17,18] or inflammatory diseases such as Helicobacter pylori infection in the stomach has been discussed. In the present case, no gastric lesions were observed after stomach examination. According to Rosenstiel et al [19] in patients with Sjögren's syndrome, the risk of developing Non-Hodgkin's lymphoma increases 44-fold and 80% of these lymphomas are of the MALT type. Our patient had no clinical evidence of Sjögren's syndrome.

According to Anacak et al [6] salivary gland MALT lymphoma is mainly a disease affecting women; in their study the ratio of females to males was 3/1. Kojima et al [20] reported a female-to-male ratio of 1.7/1.0 for primary lymphomas of the salivary glands. Kalpadakis et al [21] reported a series of 76 patients with non-gastric extra-nodal marginal zone lymphomas in which two thirds of the patients were female. The reason for this female predominance is not clear.

Radiological representation of MALT lymphomas of the parotid gland is scarce [22]. According to Corr et al [23] who presented a cohort of 10 HIV-infected children with MALT lymphomas of the parotid gland, the CT scan appearance of these lesions consisted of multiple hypoechoic solid nodules, which corresponded to hyperplastic lymphoid tissue or lymphoma. Cystic lesions (from compression of terminal parotid ducts by contiguous hyperplastic or neoplastic lymphoid tissue) and punctuate calcification, both intracystic and parenchymal may coexist. This radiologic appearance has also been described in BLL encountered in patients with AIDS [24] or Sjögren's syndrome [25].

Currently, there is controversy in the reported literature regarding the accuracy of PET-CT scan in MALT lymphomas. Elstrom et al [26] evaluated the accuracy of PET-FDG in identifying various lymphomas subtypes. According to their results, PET-FDG detected 67% of marginal zone lymphoma. Hoffmann et al [27] reported increased FDG uptake in patients with nodal marginal zone lymphoma but not in those with extranodal disease, suggesting that the FDG-avidity depends on tumor location and ⁄or the lymphoma subtype. Perry et al [28] suggested that PET -CT is a useful tool for both, initial staging and follow-up after treatment in patients with MALT lymphoma and its sensitivity depends on disease location and stage at initial diagnosis.

Most non-gastric MALT lymphomas have been noted to be indolent. Disseminated disease is relatively slow to develop in affected patients. Up to 50% of the patients with non-gastric MALT lymphoma have multiple involved sites [29]. Whether this phenomenon can be attributed to synchronous disease occurrence at multiple sites or to undetected sub-clinical disease, the mechanism of disease dissemination is unknown [30].

An association between hepatitis C virus (HCV) infection and B-cell lymphomas has previously been reported, especially in countries in which the prevalence of HCV is relatively high [31,32]. Other researchers have not found this association [33,34]. Thus, further studies are needed to define the role of HCV infection in the pathogenesis of MALT lymphoma. Rosenstiel et al [19] suggested that any patient with a cystic parotid mass must be screened for HIV infection or for Sjogren disease, because it is more likely that the cystic mass is derived from either one of these underlying diseases than to a MALT lymphoma. According to Klussmann et al [35] Epstein - Barr virus (EBV), Human herpetovirus (HHV) types -6 and -8, HCV and HIV infections have been involved in the etiology of salivary MALT lymphomas.

In our practice, clinical examination, preoperative FNA of the suspicious lesion and radiological investigation in certain cases, is part of the preoperative assessment of a suspicious parotid gland lesion. However, Ando et al [36] suggested that a parotid MALT lymphoma is hard to diagnose by fine-needle aspiration cytology.

Thieblemont et al [37] suggested that patients with localized disease generally were treated with surgery or radiotherapy. Surgery is strongly recommended as a diagnostic tool of malignant lymphoma of the parotid gland [38], since histological evaluation is essential for treatment of malignant lymphoma. Parotid surgery is positively recommended both in order to treat the tumor and to ensure histological diagnosis of the tumor for further follow-up planning. The prognosis is excellent for patients with MALT lymphoma of the parotid gland. Limited data indicate five-year survival rates of more than 80%.

Once MALT lymphoma is diagnosed, an in-depth evaluation for synchronous multi-sited involvement and disseminated disease should be undertaken before initiation of local therapy. Radiotherapy and chemotherapy should be considered in association with surgery in disseminated forms or after removal. Sarris et al suggested [38] irradiation in case of localized lesions in early stage and chemotherapy in those with advanced disease. Marioni et al [15] suggested that radiotherapy and chemotherapy should be considered in association with surgery in disseminated forms or after incomplete removal. Isobe et al [39] treated 37 patients with Stage IE extragastric MALT lymphomas with radiotherapy only. Local control was obtained in 97.3% of the patients, and progression free survival at three years was reported as 91.9%. As far as chemotherapy in patients with MALT-type lymphoma is concerned, although there is limited experience [40], clinical trials of systemic therapies are strongly advised.

Regional and distant relapses are not common in gastric MALT lymphomas, but extragastric MALT lymphomas tend to be more aggressive and may recur in the regional or distant lymph nodes and in other organs [30,41]. According to Wenzel et al, patients with MALT-lymphoma of the head and neck are at a relatively high risk for early dissemination and subsequent distant recurrence when only local therapies are applied. In the current case, there was no lymph node or other organ involvement.

## Conclusion

Salivary gland MALT lymphoma should be considered in the differential diagnosis of cystic or bilateral salivary gland lesions. After histopathological confirmation of a suspicious parotid gland lesion as a parotid gland MALT lymphoma, careful follow-up is needed with attention either to the remaining parotid gland or to other major salivary glands and organs in the head and neck region.

## Consent

Written informed consent was obtained from the patient for publication of this case report and accompanying images. A copy of the written consent is available for review by the Editor-in-Chief of this journal.

## Competing interests

The authors declare that they have no competing interests.

## Authors' contributions

PKo prepared the manuscript and reviewed it for publication. ES performed the review of the literature. PKa collected the patients' data. GK supervised the general management and follow-up of the patient and the writing of the manuscript. All authors read and approved the final manuscript.

## Abbreviations Section

MALT: mucosa associated lymphoid tissue; BLL: benign lymphoepithelial lesion
